# Developing clinical decision tools to implement chronic disease prevention and screening in primary care: the BETTER 2 program (building on existing tools to improve chronic disease prevention and screening in primary care)

**DOI:** 10.1186/s13012-015-0299-9

**Published:** 2015-08-04

**Authors:** Donna Patricia Manca, Denise Campbell-Scherer, Kris Aubrey-Bassler, Kami Kandola, Carolina Aguilar, Julia Baxter, Christopher Meaney, Ginetta Salvalaggio, June C. Carroll, Vee Faria, Candace Nykiforuk, Eva Grunfeld, and the original BETTER Trial Investigators and Clinical Working Group

**Affiliations:** Department of Family Medicine, University of Alberta, 6-10 University Terrace, Edmonton, Alberta T6G 2T4 Canada; Covenant Health, Grey Nuns Community Hospital, 1100 Youville Drive Northwest, Edmonton, Alberta T6L 5X8 Canada; Discipline of Family Medicine, Memorial University of Newfoundland, 300 Prince Phillip Drive, St. John’s, Newfoundland A1B 3V6 Canada; Department of Health and Social Services, Government of Northwest Territories, P.O. Box 1320, Yellowknife, Northwest Territories X1A 2L9 Canada; Department of Family and Community Medicine, University of Toronto, 500 University Avenue, Toronto, Ontario M5G 1V7 Canada; School of Public Health, University of Alberta, 11405 87 Avenue, Edmonton, Alberta T6G 1C9 Canada; Ontario Institute for Cancer Research, 661 University Avenue, Suite 510, Toronto, Ontario M5G 0A3 Canada

## Abstract

**Background:**

The *B*uilding on *E*xisting *T*ools *t*o Improv*e* Chronic Disease P*r*evention and Screening in Family Practice (BETTER) trial demonstrated the effectiveness of an approach to chronic disease prevention and screening (CDPS) through a new skilled role of a ‘prevention practitioner’(PP). The PP has appointments with patients 40–65 years of age that focus on primary prevention activities and screening of cancer (breast, colorectal, cervical), diabetes and cardiovascular disease and associated lifestyle factors. There are numerous and occasionally conflicting evidence-based guidelines for CDPS, and the majority of these guidelines are focused on specific diseases or conditions; however, primary care providers often attend to patients with multiple conditions. To ensure that high-level evidence guidelines were used, existing clinical practice guidelines and tools were reviewed and integrated into blended BETTER tool kits. Building on the results of the BETTER trial, the BETTER tools were updated for implementation of the BETTER 2 program into participating urban, rural and remote communities across Canada.

**Methods:**

A clinical working group consisting of PPs, clinicians and researchers with support from the Centre for Effective Practice reviewed the literature to update, revise and adapt the integrated evidence algorithms and tool kits used in the BETTER trial. These resources are nuanced, based on individual patient risk, values and preferences and are designed to facilitate decision-making between providers across the target diseases and lifestyle factors included in the BETTER 2 program. Using the updated BETTER 2 toolkit, clinicians 1) determine which CDPS actions patients are eligible to receive and 2) develop individualized ‘prevention prescriptions’ with patients through shared decision-making and motivational interviewing.

**Results:**

The tools identify the patients’ risks and eligible primary CDPS activities: the patient survey captures the patient’s health history; the prevention visit form and integrated CDPS care map identify eligible CDPS activities and facilitate decisions when certain conditions are met; and the ‘bubble diagram’ and ‘prevention prescription’ promote shared decision-making.

**Conclusion:**

The integrated clinical decision-making tools of BETTER 2 provide resources for clinicians and policymakers that address patients’ complex care needs beyond single disease approaches and can be adapted to facilitate CDPS in the urban, rural and remote clinical setting.

**Trial registration:**

The registration number of the original RCT BETTER trial was ISRCTN07170460.

**Electronic supplementary material:**

The online version of this article (doi:10.1186/s13012-015-0299-9) contains supplementary material, which is available to authorized users.

## Background

### Context

The prevalence of chronic disease is steadily increasing [1, 2], and primary care is an ideal setting for chronic disease prevention and screening (CDPS) activities [3–5]. Regrettably, evidence-based tools and strategies for CDPS are inconsistently applied in the primary care setting, in part due to the numerous and sometimes conflicting recommendations and guidelines [6, 7]. Since 45 % of people have one or more chronic disease [8], primary care providers need effective strategies that address multiple conditions. However, guidelines are focused on specific conditions or risk factors [7, 9], which makes it difficult for clinicians to address patients’ unique risk profiles. Thus, a comprehensive evidence-based approach to CDPS has been ‘lost in translation’ [10] and there is a need to engage end-users including clinicians, researchers and policymakers in a collaborative process [10] to address this knowledge to action gap. Furthermore, with the competing demands on primary care providers there is little time to address CDPS [11, 12]; hence, a new approach that bridges the evidence to practice gap in primary care CDPS is needed.

**Fig. 1:**
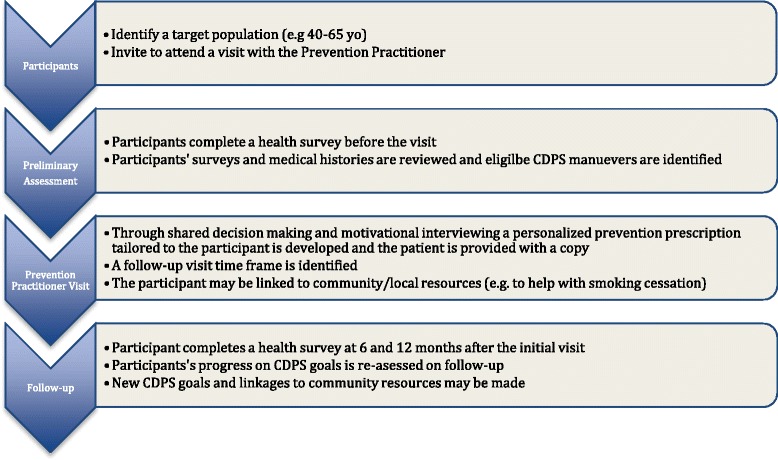
The BETTER 2 chronic disease prevention and screening prevention practitioner intervention

The *B*uilding on *E*xisting *T*ools *t*o Improv*e* Chronic Disease P*r*evention and Screening in Family Practice (BETTER) trial was a pragmatic two-way factorial cluster randomized controlled trial conducted in urban primary care team practices in Alberta and Ontario, Canada [12]. Patients aged 40–65 were invited to participate in the trial and stratified into groups: 1) general medical patients and 2) patients with moderate mental illness. The BETTER tool kit and training provided the ‘prevention practitioners’ (PP) in the two urban settings with the necessary tools and resources to evaluate patients for multiple risks. The tools were aimed to prevent multiple chronic conditions through a process of shared decision-making, which provided the patient with an individualized ‘prevention prescription’ that included actionable CDPS goals (see Fig. 1). The PPs in the BETTER trial were clinicians (licensed practical nurse, nurse, dietician, nurse practitioner) who worked in the multidisciplinary primary care clinics to develop a comprehensive approach to evidence-based CDPS within the practice setting [7, 12, 13].

To bridge the gap between knowledge and practice, a BETTER trial clinical working group (CWG) was formed with end-users that included PPs, clinicians and researchers with support from the Centre for Effective Practice [7]. The CWG identified and harmonized high-quality clinical practice guidelines and tools for primary CDPS in adults 40–65 years of age [7, 12], creating the BETTER tool kit that was implemented in the BETTER trial. This extensive review also defined the scope of the BETTER trial; the chronic diseases with the best evidence for primary prevention and screening were identified and incorporated into the comprehensive approach to CDPS. The target conditions included in the BETTER trial were cancer (breast, cervical, and colorectal), cardiovascular disease, diabetes and their associated lifestyle factors (smoking, alcohol consumption, diet/nutrition and physical activity). The BETTER trial demonstrated the effectiveness of a shared decision-making approach to CDPS that improved the implementation of clinically important CDPS activities through the new skilled role of a ‘prevention practitioner’ in both patient strata [12].

Once the BETTER trial demonstrated that a PP could improve the implementation of clinically important CDPS actions in a cost-effective manner [12], further funding was obtained to broaden the reach to other jurisdictions including urban, rural and remote communities in Canada and deepen the impact of the intervention through the *B*uilding on *E*xisting *T*ools *t*o Improv*e* Chronic Disease P*r*evention and Screening in Primary Care (BETTER 2) program [14]. To achieve this goal and bridge the evidence to practice gap, revisions to the BETTER trial kit were required in order to update the evidence and adapt the tools into a format that could be used in diverse primary care settings including rural and remote settings and with aboriginal populations. This process was, in part, informed by feedback received from the participating clinicians and patients in the BETTER trial as it indicated that some of the items could be modified or removed, while others, such as the family history, could be better formatted to improve data capture. We describe here the process of integrated knowledge translation [15–18] that involved engaging end-users from the various practice settings (including clinicians and policymakers) with researchers as equal partners in this knowledge synthesis and the development of the resulting BETTER 2 tools.

### Purpose

The purpose of this paper is to describe 1) the integrated process used to adapt and refine the BETTER trial tools for chronic disease prevention and screening (CDPS) by the BETTER 2 program and 2) the resultant tools that were then implemented into various urban, rural, remote and aboriginal primary care settings by the BETTER 2 program.

## Methods/design

**Fig. 2:**
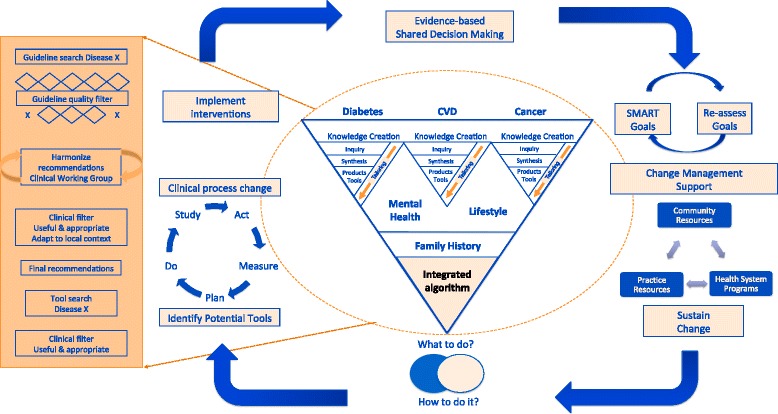
Guideline harmonization and implementation plan for the BETTER trial. The evidence integration and implementation process for the Building on Existing Tools to Improve Chronic Disease Prevention and Screening in Family Practice (BETTER) trial. The *triangle* in the centre of the diagram is an extension of the ‘knowledge creation funnel’ in the knowledge-to-action cycle [10]. In our process, there is knowledge synthesis with each funnel representing existing literature captured in high-quality clinical practice guidelines. This is then contextually integrated for each patient’s family history and modifiable risk factors. The *side bar* shows the structured evidence review process of the clinical working group. The *boxes* around the circumference of the cycle refer to the steps for implementing the recommendations and tools in both the practice- and patient-level interventions of the BETTER trial. Note: *CVD* cardiovascular disease, *EMR* electronic medical record [7]

The BETTER trial CWG identified and reviewed high-quality clinical practice guidelines and harmonized them to standardize the recommendations for implementation into the BETTER trial [7]. The CWG considered strong evidence that was linked to a target or health outcome [7] and created the knowledge products for the PP intervention that were used in the trial [7, 12]. The knowledge products were developed from November 2009 to March 2010 through a structured approach to evidence integration [7] involving a knowledge to action cycle (Fig. 2) [7, 10]. This involved engaging the researchers with end-users and policymakers in a process that included knowledge synthesis and harmonization through a structured evidence review process, and then testing and applying the tools in the various practice settings through an iterative plan-do-study-act (PDSA) process. Findings from the local PDSA activities were then integrated into the tools.

The BETTER 2 CWG was convened in November 2012; this group included end-users, clinicians (family physicians, registered nurses, nurse practitioners), policymakers from the new jurisdictions (Northwest Territories, Newfoundland and Labrador) and researchers tasked with reviewing and updating the high-quality recommendations for primary prevention of chronic conditions in patients 40–65 years of age. The chronic conditions included cardiovascular disease, diabetes and breast, colorectal, lung and cervical cancer, as well as the associated lifestyle risk factors (e.g. tobacco use, alcohol overuse, poor diet and physical inactivity) [7, 12]. A targeted search using the process described in our previous publication [7] was conducted to identify new resources meeting any of the following criteria:

1. Date of publication subsequent to 2009;
2. Addressing a gap or special population not considered in the original BETTER trial search;
3. Interventions strongly recommended for application in practice;
4. Recommendations for patients at higher risk due to family history;
5. New resources identified through scoping reviews of provincial and territorial recommendations.

The CWG was divided into teams focusing on the following conditions: breast cancer, cervical cancer, colorectal cancer, skin cancer, lung cancer, cardiovascular disease, diabetes, alcohol, mental health, lifestyle (tobacco, alcohol, nutrition and physical activity), obesity (waist circumference, BMI) and family history.

Scoping reviews of provincial and territorial recommendations were also conducted to assist with further tailoring of the tools to comply with the approaches to CDPS in participating Canadian provincial or territorial jurisdictions (Alberta, Ontario, Northwest Territories, Newfoundland and Labrador). For example, a decision was made to reduce the consumption thresholds recommended in Canada’s low-risk alcohol drinking guidelines [19] as the Northwest Territories was concerned with the high rates of colorectal cancer in their jurisdiction concomitant with heavy drinking [20] and the potential for increased cancer risk in those exceeding the alcohol levels recommended by the Canadian Cancer Society [21, 22]. The CWG concluded that the Canadian guidelines were more focused on the risk of developing an alcohol use disorder and therefore did not adequately inform individuals about lower alcohol consumption levels to reduce the risk for chronic disease such as cancer. The tools were adapted to focus on informing Canadians about safer levels of alcohol consumption to prevent chronic disease, an approach that has since been recommended by the Canadian Cancer Society [22].

The members of the BETTER 2 CWG individual topic teams met, independently reviewed and critiqued the new information identified and presented their assessments to the CWG for review by the entire group in November 2012, December 2012 and January 2013. To address gaps and build on the previous work, a greater emphasis was placed on tools that would facilitate family history assessment, address local disease and risk factor prevalence and help harness local resources. Following this, the BETTER tools were updated, reviewed, edited and tested by various members of the BETTER 2 CWG to determine if they were useful and appropriate in the various clinical settings. The review and BETTER 2 tool kit refinement was completed in January 2013 after which training sessions were held with CWG members and PPs to implement the updated tools into the various practice settings.

## Results

As a result of the comprehensive work of the BETTER 2 CWG, the tools were updated and adapted to address CDPS in the various urban, rural, remote and aboriginal contexts. The following tools were refined for inclusion in the BETTER 2 tool kit: a patient health survey (Additional files 1 and 2), a CDPS care map (Additional file 3), a prevention visit form (Additional file 4), the bubble diagrams (Additional file 5) and the prevention prescription with goals (Additional file 6). The tool kit can be accessed on the BETTER website [23]. This toolkit was the foundation for the comprehensive approach to CDPS implemented in participating jurisdictions and was further customized for each practice setting through the identification of local, regional and national resources that could be harnessed to support patients’ CDPS care plans and lifestyle change goals.

### Using the BETTER tools

The patient survey (Additional files 1 and 2) and prevention visit form (Additional file 4) capture the patient information and characteristics needed to make CDPS recommendations. The CDPS care map (Additional file 3), informed by the aforementioned data collection instruments, is a clinical decision aid that helps the clinician determine which CDPS recommendations the patient is eligible to receive when certain criteria a met, including when to refer the patient back to their primary care provider. The bubble diagrams (Additional file 5) are also instructive to both the clinician and patient as to the CDPS activities a patient is eligible to receive and can be used to facilitate agenda setting with the patient. The prevention prescription (Additional file 6) is a document intended for the patients to take with them when they leave the visit to help inform them of their prevention and screening status and guide patients on when, where and how they will go about improving deficient CDPS recommendations. Through a shared decision-making process between the clinician and patient, patients also set specific, measurable, attainable, realistic, timely (SMART) goals for their health (Additional file 6), providing the patient and clinician with a personalized plan geared toward enabling patients to achieve their CDPS goals.

### Patient survey

#### The BETTER survey

The BETTER patient health survey (Additional file 1) provides primary care providers with a tool that captures the comprehensive patient information required to facilitate CDPS and monitor progress including the important behavioural, environmental and familial risk factors. This tool comprises validated instruments (Additional file 2) that can gather detailed information relevant to CDPS including chronic conditions, previous cancer screening activities, lifestyle information and risks, perception of general health and depression, family history of certain medical conditions, food security and demographic information (age, gender, ethnicity, education, marital status, income). Much of this information is not routinely collected or available in the medical record; yet, this information is required for a clinician to determine the CDPS actions an individual patient should focus on.

The BETTER trial survey was refined for use in BETTER 2 based partly on feedback received indicating that the tool could be streamlined and reformatted to improve data capture and usability. The original patient health survey was lengthy, consisting of 88 items [12], and included an assessment of physical activity using activity recall [24] and a dietary assessment derived from the MEDFICTS, a dietary instrument with a focus primarily on fat intake [25, 26]. After review by the BETTER 2 CWG, it was determined that employment of other validated tools to assess diet and exercise, which had been developed for use in primary care, could improve the ability to identify those patients who would benefit from a brief intervention and track changes over time.

Improvements to the original survey were made to better capture physical activity assessments including use of the general practice physical activity questionnaire (GPPAQ) [27] to determine the patients’ level of activity in addition to the patients’ self-reported number of minutes spent on exercise weekly to determine if patients are achieving a CDPS target of ≥150 min per week of moderate exercise [28–32]. The GPPAQ is a reliable and validated tool recommended for the assessment of physical activity in general practice that is supported by the United Kingdom National Institute for Health and Care Excellence (NICE) [27]. This tool assesses the respondent’s level of physical activity both in and outside of work and can be used by clinicians to inform when an intervention to increase physical activity would be beneficial as well as to track a patient’s progress over time.

A validated tool for dietary assessment and intervention in the clinical setting, Starting the Conversation [33], was added to provide the clinician with insight into patients’ eating behaviours and information on how patients could improve their diet (e.g. increase fruit and vegetables, decrease sweetened beverages, decrease unhealthy snacks).

Alcohol consumption is now captured quantitatively to determine if patients are drinking within healthy alcohol consumption guidelines according to the National Institute on Alcohol Abuse and Alcoholism’s overview of alcohol consumption for low-risk drinking [34]. Alcohol use disorders are also screened for using the validated abbreviated form of the alcohol use disorders identification test, the AUDIT-C [35–38]. The updated tools provide clinicians with an approach that educate patients about healthy alcohol consumption as opposed to only screening for abuse.

The health survey was reduced from 88 to 69 items to more efficiently capture information on the important modifiable lifestyle risk factors (smoking, alcohol consumption, diet/nutrition and physical activity) and includes assessments of the patient’s readiness to change, gathering the information required to address these risk factors [39, 40]. The health survey can take up to 30 min to complete. It may be completed before the patient’s prevention visit either independently by the patient or administered by a health care professional when deemed appropriate (e.g. literacy, language).

### Prevention visit form

The prevention visit form (Additional file 4) is a clinical tool that captures and structures the information obtained from the patient’s survey and medical chart required to identify which prevention activities each patient is (or is not) eligible to receive. Typically, the clinician will partially complete this form before the patient visit to identify the CDPS activities eligible for discussion. Further information is collected at the time of the prevention visit including a limited physical assessment of the patient to obtain weight, height, waist circumference and blood pressure. Before the patient visit, the clinician can enter the patient’s individual CDPS information on a blank version of the bubble diagram (Additional file 5) and the first page of the prevention prescription (Additional file 6).

### CDPS care maps

The CDPS care map (Additional file 3) provides primary care providers with an algorithm of the summarized CDPS recommendations for primary prevention in 40–65 year olds for patients with and without diabetes. A health care provider can use the care map as a decision-making tool during the prevention visit to determine what actions to take when certain conditions are met. This includes consideration as to what CDPS actions a patient is eligible or not eligible to receive, and when to refer a patient back to their primary care provider. For some actions, particularly cardiovascular related CDPS, recommendations depend upon whether a patient has diabetes or not. Use of the CDPS care map is facilitated by information gathered from the patient survey, the prevention visit form and during the prevention visit. Other tools such as the Framingham risk stratification and/or a family history risk assessment tool can also be used to provide further information about the patient’s risks of diabetes, cardiovascular disease or cancer.

### Bubble diagram

The bubble diagram (Additional file 5) provides a brief overview of the blended evidence-based CDPS activities for primary prevention in 40–65 year old male and female patients. Regular screening intervals and healthy targets summarized in this tool are meant as a companion piece to the CDPS care map, which depicts the appropriate care path for patients depending on their level of risk. Specific patient details can be entered on a blank version of the bubble diagram and then used as a teaching tool when meeting with patients. The bubble diagrams can facilitate a motivational interviewing approach through agenda setting [39]. For example, after educating the patient about CDPS and while showing the patient their individualized bubble diagram, the clinician can ask the patient what they want to do to improve their health and begin the work that is finalized in the prevention prescription (described below). Intrinsic in the patient-centred approach is the ability of the patient to opt out of discussing any area that they do not wish to address. The bubble diagram allows the negotiation of a shared agenda for the prevention visit through a visual emphasis on the bubbles the patient wants to address.

The bubble diagram with the evidence overview can also be used as a visual cue to remind primary care providers about the CDPS activities to consider when seeing patients in this age group.

### Prevention prescription with goals

The prevention prescription (Additional file 6) includes a summary of the patient’s CDPS status, target check-in intervals, referrals or actions to be completed, and any tools provided or linkages made to clinic or community resources to aid the patient in their CDPS efforts. The information on the prevention prescription that does not require shared decision-making can be partially completed before the visit and then finalized with input from the patient at the time of the visit. The goal sheet facilitates shared decision-making through the development of SMART goals including an assessment of confidence addressing action planning and self-efficacy in patient self-management [41, 42]. The prevention prescription with goals can be provided to the patient as a summary of their visit and serve as the patient’s personalized CDPS plan.

## Discussion

### The need for a BETTER approach to CDPS

Busy clinicians lack adequate tools and resources to address CDPS in the primary care setting since many guidelines focus on specific conditions [7, 9] and lack precise recommendations that are clinically applicable [7]. The BETTER tools bridge the knowledge to action gap through a blended approach of actionable items at every step in the process of CDPS from collecting the necessary patient information to care maps for primary care providers and teams [7]. Through engaging the end-users in the process of developing CDPS tools and resources and applying the tools into the clinical setting, the BETTER trial was able to effectively implement CDPS in the family practice setting through a new skilled role of ‘prevention practitioner’ (PP) [12]. These tools and resources further facilitate knowledge uptake by patients through agenda setting, shared-decision-making and self-management. The updated tools and resources described in this paper were refined in order to further facilitate patient assessments, education and shared decision-making aimed to identify and achieve the patient’s personalized CDPS goals as well as capture the information required to evaluate CDPS outcomes for the BETTER 2 program implementation [14] in urban, rural, remote and aboriginal settings.

Modifiable lifestyle factors such as smoking, unhealthy eating, physical inactivity and unhealthy alcohol consumption have a huge impact on chronic disease [43] and there is a pressing need to address multiple behavioural risk factors in primary care [44]. Although addressing modifiable lifestyle risk factors can significantly impact mortality and morbidity [43, 45] few individuals receive lifestyle counselling, even after a significant illness such as a cardiovascular event [46, 47]. The updated BETTER 2 tools provide clinicians with resources that evaluate the patient’s multiple lifestyle risks including their readiness to change and can be used to track changes over time. In addition, these tools facilitate shared decision-making with patients through agenda setting and identifying specific goals that encourage self-management [41, 42].

**Fig. 3:**
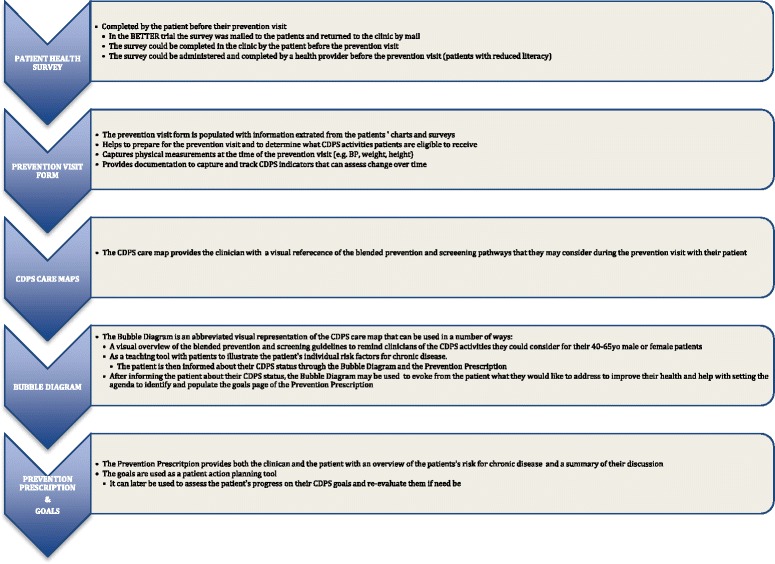
The BETTER tools

The tools are tailored to be adaptable and can be used in a number of ways as depicted in Fig. 3. In this way, multidisciplinary teams or family physicians can decide how best to apply the tools in their clinical settings. The updated BETTER approach may be used at the policy and practice level to target at risk populations and invite patients to receive an effective individualized CDPS intervention based on high-level evidence [12] supported by the BETTER 2 tool kit. Moreover, the tools can be harmonized and integrated with existing public health initiatives in various settings. For example, an initiative involving population or practice level CDPS facilitation may consider the BETTER approach or integrating some of the tools to translate population level CDPS activities to individual at-risk patients. Policymakers may provide primary care providers with the BETTER tools to better implement CDPS into practice. Decision makers and primary care providers may adapt the tools to facilitate policy and practice integration of CDPS including consistent messages at all levels.

This approach to knowledge integration is not without its limitations. The tools and resources developed focus on primary prevention of CDPS in patients aged 40–65; hence other high-level interventions such as immunizations, secondary prevention and chronic disease management are not included. The BETTER tools and resources were developed with knowledge integration considered at every step to bridge the research to practice gap through an implementation plan that engaged the end-users and applied the developed resources into the practice setting (Fig. 2). Consequently, the final tools may not reflect the ‘best’ evidence but the best evidence that could be applied into the settings engaged. Also, over time, the various guidelines change, requiring constant updating and revisions to the tools. Furthermore, the process of knowledge integration is time consuming and requires organization, expertise and resources to conduct. The time and resources required to integrate knowledge into practice settings are not readily available in a health care system that is designed to focus on acute medicine and disease management. In addition to developing clinical practice guidelines, resources should also be used to develop and refine tools and processes, such as the BETTER 2 tools that allow guidelines to be more easily implemented into clinical practice.

The BETTER 2 tools have been implemented and tested in the various practice settings [14] and the outcomes will be presented in a future publication. The BETTER 2 tools can be downloaded for use from the BETTER website [23]. Presently, the tools have been paper based which may limit the ability to implement the PP model into primary care in Canada due to the increased use of electronic medical records in primary care. Electronic versions of the patient survey and prevention prescription are currently being developed and will be tested in primary care settings in Alberta, Newfoundland and Labrador.

Primary care teams should consider implementing the PP role to better address CDPS in conjunction with the primary care provider and thereby share the management of chronic disease prevention and screening. In Canada, prevention and early detection could reduce the burden of managing acute and chronic conditions. Despite that, there are barriers to CDPS. In many jurisdictions, the fee-for-service system does not remunerate prevention activities. Also, some settings lack the clinical resources to address the acute and chronic medical needs of the community. Hence there is limited capacity to implement the PP model in settings that do not compensate for this CDPS or that lack clinical resources to address and manage the acute and chronic conditions. The process could be implemented in other countries with heath care systems that have the resources to support this type of activity.

## Conclusion

The BETTER tools are a first step to structure CDPS in primary care in a comprehensive, structured, personalized and evidence-based manner and to improve the application of knowledge into practice. The integrated clinical decision-making tools of BETTER 2 provide a resource for clinicians and policymakers that address patients’ complex care needs beyond a single disease approach and can be adapted to facilitate CDPS in various primary care clinical setting in urban, rural and remote communities.

## Additional files

Additional file 1:
**Patient health survey.** (PDF 551 kb) Additional file 2:
**Components and sources of the patient health survey.** (DOCX 111 kb) Additional file 3:
**CDPS care maps.** (PDF 184 kb) Additional file 4:
**Prevention visit form.** (PDF 494 kb) Additional file 5:
**The Bubble diagram.** (PDF 934 kb) Additional file 6:
**The prevention prescription.** (PDF 354 kb)

## Abbreviations

BETTER trial: *B*uilding on *E*xisting *T*ools *t*o Improv*e* Chronic Disease P*r*evention and Screening in Family Practice
BETTER 2: *B*uilding on *E*xisting *T*ools *t*o Improv*e* Chronic Disease P*r*evention and Screening in Primary Care
CDPS: chronic disease prevention and screening
CLASP: Coalitions Linking Action and Science for Prevention
CPAC: Canadian Partnership Against Cancer
CWG: clinical working group
PP: prevention practitioner
SMART: specific, measurable, attainable, realistic, timely
