# Supplementary material for: Developing clinical decision tools to implement chronic disease prevention and screening in primary care: the BETTER 2 program (building on existing tools to improve chronic disease prevention and screening in primary care)
Source: Implement Sci. 2015 Aug 4;10:107. doi: 10.1186/s13012-015-0299-9 (PMC4523915; doi:10.1186/s13012-015-0299-9)
Supplement: Additional file 1: — Patient health survey. (PDF 551 kb) [file 13012_2015_299_MOESM1_ESM.pdf]

Patient Initials: ____|____|____

Date completed: _______/_______/_______
(month) (day) (year)

BETTER

Health Survey

First Visit

Thank you for completing the BETTER Health Survey!

Your answers will help us provide better care for you.

During the visit, you and your healthcare provider will discuss how you can improve your health and the screening tests you are eligible to receive. You will be able to set your own health goals and create a plan that will help you achieve them.

Later, your healthcare provider will check with you to review your progress, help you change your plan, if needed, and set new goals.

INSTRUCTIONS: Please answer all questions to the best of your ability.

For each of the questions, please circle the number that matches your answer or fill in the blank as needed.

Your answers will help your healthcare provider prepare for your Prevention visit.

You are free to refuse to answer any question you wish.

If you wish to make a comment on any of the questions, please use the space in the margins.

Please complete your survey immediately and return it to your healthcare provider.

THE BETTER PROGRAM

SECTION A: GENERAL INFORMATION

Instructions: These questions are for both men and women.

For each of the following questions, please circle the number that corresponds to your response or fill in the blank as appropriate.

1. **Gender:** (Circle one number)

1. Male
2. Female

2. **What is your date of birth?** ______ (month) ______ (day) ______ (year)

e.g. Feb 02 1959

SECTION B: CHRONIC HEALTH CONDITIONS

Instructions: These questions are for both men and women.

The next questions ask about some health conditions that you may have that were diagnosed by a healthcare professional.

3. As far as you know, do you presently have Diabetes? (Circle one number)

1. Yes
2. No → go to question 4

3a. **What type of diabetes were you diagnosed with?** (Circle one number)

1. Type 1 (formerly juvenile diabetes; insulin-dependent diabetes)
2. Type 2 (formerly non-insulin dependent diabetes)
3. Gestational diabetes

Have you ever had:

4. **A stroke?** (Circle one number)

1. Yes
2. No

5. Heart trouble (such as angina, heart attack, congestive heart failure, or coronary artery disease)? (Circle one number)

1. Yes
2. No

SECTION C: COLORECTAL (BOWEL) SCREENING

Instructions: These questions are for both men and women.

For each of the following questions, please circle the number that corresponds to your response or fill in the blank as appropriate.

6. Have you had colorectal cancer? (Circle one number)

1. Yes → go to Section D (next page)
2. No

7. Have you ever had a fecal occult blood test (or FOBT), fecal immunochemical test (or FIT), colonoscopy, sigmoidoscopy or other test for colorectal cancer? An FOBT is a test to check for blood in your stool, where you have a bowel movement and use a stick to smear a small sample on a special card. A FIT is a test to check for blood in your stool, where you have a bowel movement and use a swab to collect a sample. A colonoscopy or sigmoidoscopy is a test where a long tube is inserted into the rectum to view the bowel for early signs of cancer and other health problems. (Circle one number)

1. Yes
2. No → go to Section D (next page)
3. Don't know → go to Section D (next page)

8. When was the last time you had an FOBT or FIT? (Circle one number)

1. Less than 6 months ago
2. 6 months to less than 1 year ago
3. 1 year to less than 2 years ago
4. 2 years to less than 3 years ago
5. 3 or more years ago
6. Don't know

9. **When was the last time you had a colonoscopy, sigmoidoscopy or other test for colorectal cancer?** (Circle one number)

1. Less than 2 years ago
2. 2 years to less than 5 years ago
3. 5 years to less than 8 years ago
4. 8 years to less than 10 years ago
5. 10 or more years ago
6. Don't know

SECTION D: SCREENING TESTS AND CHRONIC HEALTH CONDITIONS FOR WOMEN

Instructions: These questions are for women. If you are male, please go to Section E (page 6).

For each of the following questions, please circle the number that corresponds to your response or fill in the blank as appropriate.

Have you ever had:

10. **Cervical cancer?** (Circle one number)

1. Yes → go to question 15
2. No

11. **A hysterectomy?** (Circle one number)

1. Yes → go to question 15
2. No

12. **Have you had a Pap test in the past 3 years?** (Circle one number)

1. Yes
2. No → go to question 15
3. Don't know → go to question 15

13. When was the last time you had a Pap test? (Circle one number)

1. Less than 6 months ago
2. 6 months to less than 1 year ago
3. 1 year to less than 2 years ago
4. 2 years to less than 3 years ago
5. 3 or more years ago
6. Don't know

14. Were any of the Pap tests in the last 3 years abnormal? (Circle one number)

1. Yes
2. No
3. Don't know

Have you ever had:

15. Ovarian cancer? (Circle one number)

1. Yes
2. No

16. Breast cancer? (Circle one number)

1. Yes → go to Section E (next page)
2. No

17. A bilateral mastectomy? (Circle one number)

1. Yes → go to Section E (next page)
2. No

18. When was the last time you had a mammogram? A mammogram is a low-dose x-ray of the breast. (Circle one number)

1. Less than 6 months ago
2. 6 months to less than 1 year ago
3. 1 year to less than 2 years ago
4. 2 years to less than 3 years ago
5. 3 or more years ago
6. Don't know

SECTION E: MEDICATIONS

Instructions: These questions are for both men and women.

The table below lists health issues that people commonly take prescriptions for.

19. For each health issue in the table below, please circle the number that best indicates if you a) have ever been prescribed any medications and b) are currently taking any prescription medications.

Health Issues	a) Have you ever been prescribed medications for:			b) Are you currently taking prescription medication for:		
	Yes	No	Don't know	Yes	No	Don't know
i. Blood pressure	1	2	3	1	2	3
ii. Diabetes	1	2	3	1	2	3
iii. Cholesterol	1	2	3	1	2	3
iv. Smoking cessation	1	2	3	1	2	3
v. Alcohol cessation	1	2	3	1	2	3
vi. Mental Health (e.g. anxiety, depression, etc.)	1	2	3	1	2	3

SECTION F: LIFESTYLE AND NUTRITION

Instructions: These questions are for both men and women.

The next group of questions ask about some of your lifestyle behaviours such as smoking, physical activity, eating habits and alcohol consumption.

For each of the following questions, please circle the number that corresponds to your response or fill in the blank as appropriate.

20. Do you currently smoke cigarettes every day, some days, or not at all? (Circle one number)

1. Every day
2. Some days
3. Not at all → go to question 25

21. On average, how many cigarettes do you currently smoke each day?

a. Number of cigarettes smoked daily: ________.

22. Are you prepared to decrease the number of cigarettes you currently smoke? (Circle one number)

1. Yes
2. No → go to question 25

23. Please indicate how prepared you are to decrease the number of cigarettes you currently smoke. Where 0 = not prepared to decrease and 10 = already decreasing. (Circle one number)

How prepared

**0**	1	2	3	4	5	6	7	8	9	**10**
**Not prepared to decrease**										**Already decreasing**

24. How confident are you that you can decrease the number of cigarettes you currently smoke? Where 0 = not at all confident and 10 = extremely confident. (Circle one number)

How confident

**0**	1	2	3	4	5	6	7	8	9	**10**
**Not at all confident**										**Extremely confident**

25. In the past six months have you exercised regularly (at least once per week)? Exercise is defined as any activity that requires physical effort, increases your heart rate and may cause shortness of breath. (Circle one number)

1. Yes
2. No → go to question 27

26. In a typical week, how many minutes do you spend exercising?

a. Number of minutes spent exercising weekly: ________.

27. Please tell us the type and amount of physical activity involved in your work. (Circle one number)

1. I am not employed (for example: retired, retired for health reasons, unemployed, full-time carer, etc.)
2. I spend most of my time at work sitting (such as in an office)
3. I spend most of my time at work standing or walking. However, my work does not require much intense physical effort (for example: shop assistant, hairdresser, security guard, child care provider, etc.)
4. My work involves definite physical effort including handling of heavy objects and use of tools (for example: plumber, electrician, carpenter, cleaner, hospital nurse, gardener, postal delivery workers, etc.
5. My work involves vigorous physical activity including handling of very heavy objects (for example: scaffolder, construction worker, refuse collector, etc.)

28. During the last week, please indicate with an "x" how many hours you spent on each of the following activities. Please answer the questions even if you are not employed.

	None	Some, but less than 1 hour	1 hour, but less than 3 hours	3 hours or more
a) Physical exercise such as swimming, jogging, aerobics, football, tennis, gym workout, etc.				
b) Cycling, including to work and during leisure time.				
c) Walking, including walking to work, shopping, for pleasure, etc.				
d) Housework/Childcare				
e) Gardening/Do-it-yourself projects				

29. How would you describe your usual walking pace? (Circle one number)

1. Slow pace
2. Steady average pace
3. Brisk pace
4. Fast pace

30. **Are you prepared to increase the amount of exercise you currently do?** (Circle one number)

1. Yes
2. No → go to question 33

31. **Please indicate how prepared you are to increase the amount of exercise you currently do.** Where 0 = not prepared to increase and 10 = already increasing. (Circle one number)

How prepared

**0**	1	2	3	4	5	6	7	8	9	**10**
**Not prepared to increase**										**Already increasing**

32. **How confident are you that you can increase the amount of exercise you currently do?** Where 0 = not at all confident and 10 = Extremely confident. (Circle one number)

How confident

**0**	1	2	3	4	5	6	7	8	9	**10**
**Not at all confident**										**Extremely confident**

33. **In general, how would you rate your eating habits?** (Circle one number)

1. Excellent
2. Very Good
3. Good
4. Fair
5. Poor

Please tell us, over the past few months:

34. **How many times a week did you eat fast food meals or snacks?** (Circle one number)

1. Less than 1 time
2. 1 – 3 times
3. 4 or more times

35. How many servings of fruit did you eat each day? (Circle one number)

1. 5 or more
2. 3 – 4
3. 2 or less

36. How many servings of vegetables did you eat each day? (Circle one number)

1. 5 or more
2. 3 – 4
3. 2 or less

37. How many regular sodas, glasses of sweet tea or sugar sweetened beverages did you drink each day? (Circle one number)

1. Less than 1
2. 1 – 2
3. 3 or more

38. How many times a week did you eat regular snack chips or crackers (not low-fat)? (Circle one number)

1. 1 time or less
2. 2 – 3 times
3. 4 or more times

39. How many times a week did you eat desserts and other sweets (not the low-fat kind)? (Circle one number)

1. 1 time or less
2. 2 – 3 times
3. 4 or more times

40. How much margarine, butter or meat fat do you use to season vegetables or put on potatoes, bread or corn? (Circle one number)

1. Very little
2. Some
3. A lot

41. Are you prepared to improve your current diet? (Circle one number)

1. Yes
2. No → go to question 44

42. **Please circle the number that shows how prepared you are to improve your current diet.** Where 0 = not prepared to improve and 10 = already improving. (Circle one number)

How prepared

**0**	1	2	3	4	5	6	7	8	9	**10**
**Not prepared to improve**										**Already improving**

43. **How confident are you that you could improve your current diet?** Where 0 = not at all confident and 10 = Extremely confident. (Circle one number)

How confident

**0**	1	2	3	4	5	6	7	8	9	**10**
**Not at all confident**										**Extremely confident**

44. **How often do you have a drink containing alcohol (e.g. beer, wine, spirits or other alcoholic beverages)?** (Circle one number)

1. Never → go to Section G (page 13)
2. Monthly or less
3. 2 – 4 times per month
4. 2 – 3 times per week
5. 4 or more times per week

45. **How many drinks containing alcohol do you have on a typical day when you are drinking?** A drink is defined as a 341 ml (12 oz.) glass containing 5% alcohol (beer, cider or cooler), 142 ml (5 oz.) glass of wine with 12% alcohol content or a 43 ml (1.5 oz.) shot/serving of 40% distilled alcohol content (rye, gin, rum, etc.) (Circle one number)

1. 1
2. 2
3. 3
4. 4
5. 5
6. 6
7. 7
8. 8
9. 9
10. 10 or more

46. How often do you have 6 or more drinks containing alcohol on one occasion? (Circle one answer)

1. Never
2. Less than monthly
3. Monthly
4. Weekly
5. Daily or almost daily

47. On average, how many drinks containing alcohol do you currently have <u>each week</u>?

a. Number of drinks containing alcohol you have weekly: ________.

48. Are you prepared to reduce the amount of alcohol you currently drink? (Circle one number)

1. Yes
2. No → go to Section G (next page)

49. Please indicate how prepared you are to reduce the amount of alcohol you currently drink. Where 0 = not prepared to reduce and 10 = already reducing. (Circle one number)

<u>How prepared</u>

**0**	1	2	3	4	5	6	7	8	9	**10**
**Not prepared to reduce**										**Already reducing**

50. How confident are you that you could reduce the amount of alcohol you currently drink? Where 0 = not at all confident and 10 = extremely confident. (Circle one number)

<u>How confident</u>

**0**	1	2	3	4	5	6	7	8	9	**10**
**Not at all confident**										**Extremely confident**

SECTION G: GENERAL HEALTH

Instructions: These questions are for both men and women.

These next questions ask for your views about your health. This information will help keep track of how you feel and how well you are able to do your usual activities.

Please circle the number that you think best describes how you feel. If you are unsure about how to answer a question, please give the best answer you can.

51. In general, would you say your health is: (Circle one number)

1. Excellent
2. Very Good
3. Good
4. Fair
5. Poor

Over the <u>past two weeks</u>, how often have you been bothered by any of the following problems?

52. Little interest or pleasure in doing things (Circle one number)

1. Not at all
2. Several Days
3. More than half the days
4. Nearly every day

53. Feeling down, depressed or hopeless (Circle one number)

1. Not at all
2. Several Days
3. More than half the days
4. Nearly every day

SECTION H: FAMILY MEDICAL HISTORY

Instructions: These questions are for both men and women.

Knowing your family history can help your health care practitioners provide better care for you.

54. The 2 tables below list some chronic diseases. Please complete the 2 tables to the best of your knowledge.

*For each of the following, please only include **blood relatives**. That is, relatives that are related to you by **blood or birth** and do not include relatives related to you by marriage.*

If one or more of your blood relatives has been diagnosed with the disease, please indicate the age of the relative who was diagnosed the youngest. For example, if your sister was diagnosed with diabetes at age 20 and your brother was also diagnosed with diabetes at age 35, you would write "20" under youngest age at diagnosis.

**First Degree Relatives**						
**Disease**	**Parents**		**Brothers or Sisters**		**Children**	
	Number of parents with disease	Youngest age at diagnosis	Total number of brothers or sisters with disease	Youngest age at diagnosis	Total number of children with disease	Youngest age at diagnosis
a. Diabetes						
b. Breast Cancer						
c. Colorectal Bowel Cancer						
d. Ovarian Cancer						
e. Cardiovascular/ Heart Disease						

Second Degree Relatives<br>*Uncles, Aunts, Nieces, Nephews and Grandparents*				
**Disease**	**On Your Mother's side**		**On Your Father's side**	
	Total number of relatives with disease	Youngest age at diagnosis	Total number of relatives with disease	Youngest age at diagnosis
f. Diabetes				
g. Breast Cancer				
h. Colorectal Bowel Cancer				
i. Ovarian Cancer				
j. Cardiovascular/ Heart Disease				

SECTION I: ABOUT YOU

Instructions: These questions are for both men and women.

For each of the following questions, please circle the number that corresponds to your response or fill in the blank as appropriate.

55. Were you born a Canadian Citizen? (Circle one number)

1. Yes → go to question 57
2. No

56. In what year did you first come to Canada to live? ____________ (year)

57. Which of the following best describes your ethnic/cultural background? (Circle one number)

1. Caucasian
2. Southeast Asian (e.g. Cambodian, Indonesian, Laotian, Vietnamese, etc.)
3. Chinese
4. South Asian (e.g. East Indian, Pakistani, Sri Lankan, etc.)
5. Filipino
6. Japanese
7. Korean
8. Latin American
9. Caribbean
10. Black/African American
11. Arab
12. West Asian (Afghan, Iranian, etc.)
13. Ashkenazi Jewish
14. Aboriginal → go to question 57a.
15. Other (Please specify): ______________________________

57a. If you are Aboriginal, please identify your Aboriginal group. (Circle one number)

1. Inuit
2. Métis
3. Innu
4. North American Indian

58. What is your highest level of education? (Circle one number)

1. Elementary school or less
2. Some high school
3. Completed high school
4. Some community college or technical school
5. Completed college or technical school
6. Some university
7. Completed bachelor's degree
8. Graduate or professional degree

59. What is your current employment status? Please circle the number that best describes your current situation. If you are self-employed, choose full-time or part-time as appropriate.

1. Employed full-time (30 hours or more a week)
2. Employed part-time (less than 30 hours per week)
3. Unable to work because of sickness or disability
4. Looking after home and/or family
5. Student
6. Retired
7. Unemployed
8. Doing unpaid or voluntary work

60. What is your marital status? Please circle the number of the status that best describes your current situation.

1. Married
2. Common-law
3. Living with a partner
4. Divorced
5. Widowed
6. Separated
7. Single or never married

61. What was your approximate total household income (from all sources) before taxes last year? (Circle one number)

1. Less than $10,000
2. $10,000 to $19,999
3. $20,000 to $39,999
4. $40,000 to $59,999
5. $60,000 to $79,999
6. $80,000 to $99,999
7. $100,000 to $124,999
8. $125,000 to $149,999
9. $150,000 to $174,999
10. $175,000 to $199,999
11. $200,000 or more

62. Which of the following statements best describes the food eaten in your household in the past 12 months? (Circle one number)

1. You and other household members always had enough of the kinds of foods you wanted to eat
2. You and other household members had enough to eat, but not always the kinds of food you wanted
3. Sometimes you and other household members did not have enough to eat
4. Often you and other household members didn't have enough to eat

The following statements may describe the food situation in a household. Please circle the number that best describes how true the statement is for your household.

63. In the past 12 months, the food that you and other household members bought just didn't last and there wasn't any money to get more.

1. Often true
2. Sometimes true
3. Never true

64. In the past 12 months, you and other household members couldn't afford to eat balanced meals.

1. Often true
2. Sometimes true
3. Never true

65. In the past 12 months, did you or other adults in your household ever cut the size of your meals or skip meals because there wasn't enough money for food? (Circle one number)

1. Yes
2. No

If you answered:

"Often true or sometimes true" to question 63 OR
"Often true or sometimes true" to question 64 OR
"Yes" to question 65
} → go to question 66

"Never true" to question 63 AND
"Never true to question 64 AND
"No" to question 65
} → go to question 69

66. How often did this happen? (Circle one number)

1. Almost every month
2. Some months but not every month
3. Only 1 or 2 months

67. In the past 12 months, did you ever eat less than you felt you should because there wasn't enough money to buy food? (Circle one number)

1. Yes
2. No

68. In the past 12 months, were you ever hungry, but didn't eat because you couldn't afford enough food? (Circle one answer)

1. Yes
2. No

69. If there is anything else that you would like to comment on, or feel is important to include, please feel free to write in the space below

____

____

____

____

____

____

____

____

____

____

____

THANK YOU for completing the BETTER Health Survey!
