# Supplementary material for: Developing clinical decision tools to implement chronic disease prevention and screening in primary care: the BETTER 2 program (building on existing tools to improve chronic disease prevention and screening in primary care)
Source: Implement Sci. 2015 Aug 4;10:107. doi: 10.1186/s13012-015-0299-9 (PMC4523915; doi:10.1186/s13012-015-0299-9)
Supplement: Additional file 2: — Components and sources of the patient health survey. (DOCX 111 kb) [file 13012_2015_299_MOESM2_ESM.docx]

**Appendix 2 - Components and sources of the patient health survey**

The **BETTER Health Survey (first visit)** contains 69 items (many of which include one or more sub questions) and 9 sections (see below) including validated tools that can be used to capture and quantify lifestyle risk factors. Space is provided for participants at the end of the survey to capture comments.

The ***General Information*** section includes 2 single-item questions about gender, and age.

The ***Chronic Health Conditions*** section includes 3 questions for both men and women about whether or not they currently have diabetes (with 1 sub question) or any history of stroke and/or heart trouble.

The ***Screening Tests*** sections include 4 single-item questions for both men and women related to colorectal cancer screening and an additional 9 single-item questions for women related to cervical, and breast cancer screening. These questions are taken or modified from the Ontario Health Survey (OHS)[[1](#_ENREF_1)] with 4 single-item questions that were developed specifically for use by the BETTER project (whether the patient has ever been diagnosed with breast, colorectal, ovarian or cervical cancer) to help determine patients’ eligibility for primary prevention and screening of these conditions..

The ***Medications*** section includes 2 questions, 1 single-item question if the patient was ever prescribed medications and 1 question about whether the patient is taking medications for blood pressure, diabetes, cholesterol, smoking cessation, alcohol cessation or mental health.

The ***Lifestyle and nutrition*** section includes 31 questions related to smoking (5 questions), exercise (8 questions) including the ‘general practice physical activity questionnaire’ (GPPAQ)[[2](#_ENREF_2), [3](#_ENREF_3)], diet (11 questions) including (7 questions) a dietary assessment tool ‘starting the conversation’ (7 questions) [[4](#_ENREF_4)], and alcohol consumption (7 questions) including the abbreviated alcohol use disorders identification test[[5](#_ENREF_5), [6](#_ENREF_6)]. The additional questions included in this section are taken or modified from the Canadian Community Health Survey CCHS[[7](#_ENREF_7)], Rollnick’s Readiness Ruler[[8](#_ENREF_8)], and a single-item from the Stanford Patient Education Centre’s “Self-efficacy for Managing Chronic Disease 6-item scale”[[9](#_ENREF_9)].

The ***General Health*** section includes 3 questions related to measuring general health, and a screen for depression using the Patient Health Questionnaire-2[[10](#_ENREF_10)].

The ***Family Medical History*** section includes 2 tables designed to collect information about the illness history (diabetes, breast cancer, colorectal cancer, ovarian cancer) of various family members (first degree and second degree relatives). These questions have been developed specifically for use by BETTER and are derived loosely from the “My Family Health Portrait” tool[[11](#_ENREF_11), [12](#_ENREF_12)] and minimum family history data recommended in the literature[[13](#_ENREF_13), [14](#_ENREF_14)].

The **About You** section includes 8 single-item demographic questions including citizenship (2 questions), ethno-cultural background, education level, employment status, marital status, and household income. These questions are taken or modified from the CCHS[[7](#_ENREF_7)], Canadian Census[[15](#_ENREF_15)], and OHS[[1](#_ENREF_1)]. Household food security is assessed in this section using the 7questions (1 lead-in question and a 6-question food security scale) which are based on the short form of the 12-month Food Security Scale created by the United States Department of Agriculture (USDA). An adapted version of the USDA’s food security scale is also used in the CCHS as part of their Household Food Security Survey Module.

References for Appendix 2

1. **The Ontario Health Survey: 1996/97 & 1990. Toronto: Association of Public Health Epidemiologists in Ontario. (Accessed August 14, 2012, at** [http://www.apheo.ca/index.php?pid=211 - 96OHS.)](http://www.apheo.ca/index.php?pid=211#96OHS.)).

2. Heron N, Tully MA, McKinley MC, Cupples ME: **Physical activity assessment in practice: a mixed methods study of GPPAQ use in primary care**. *BMC family practice* 2014, **15**:11.

3. Physical Activity Policy HID: **The general practice physical activity questionnaire (GPPAQ) - A screening tool to assess adult physical activity levels, within primary care.** In*.*; 2009.

4. Paxton AE, Strycker LA, Toobert DJ, Ammerman AS, Glasgow RE: **Starting the conversation performance of a brief dietary assessment and intervention tool for health professionals**. *Am J Prev Med* 2011, **40**(1):67-71.

5. Bush K, Kivlahan DR, McDonell MB, Fihn SD, Bradley KA: **The AUDIT alcohol consumption questions (AUDIT-C): an effective brief screening test for problem drinking. Ambulatory Care Quality Improvement Project (ACQUIP). Alcohol Use Disorders Identification Test**. *Arch Intern Med* 1998, **158**(16):1789-1795.

6. Bradley KA, Bush KR, Epler AJ, Dobie DJ, Davis TM, Sporleder JL, Maynard C, Burman ML, Kivlahan DR: **Two brief alcohol-screening tests From the Alcohol Use Disorders Identification Test (AUDIT): validation in a female Veterans Affairs patient population**. *Arch Intern Med* 2003, **163**(7):821-829.

7. **Canadian Community Health Survey (CCHS) - Cycle 1.1. Ottawa: Statistics Canada. (Accessed August 14, 2012, at** <http://www.statcan.gc.ca/concepts/health-sante/index-eng.htm.)>**.**

8. Rollnick S, Butler C, Mason P: **Health behavior change : a guide for practitioners**. Edingurgh ; Toronto: Churchill Livingstone; 1999.

9. Lorig KR, Sobel DS, Ritter PL, Laurent D, Hobbs M: **Effect of a self-management program on patients with chronic disease**. *Effective clinical practice : ECP* 2001, **4**(6):256-262.

10. Kroenke K, Spitzer RL, Williams JB: **The Patient Health Questionnaire-2: validity of a two-item depression screener**. *Med Care* 2003, **41**(11):1284-1292.

11. My Family Health Portrait: A tool from the Surgeon General. Maryland: National Institutes of Health AA, 2012, at https://familyhistory.hhs.gov/fhh-web/home.action.).

12. Facio FM, Feero WG, Linn A, Oden N, Manickam K, Biesecker LG: **Validation of My Family Health Portrait for six common heritable conditions**. *Genetics in medicine : official journal of the American College of Medical Genetics* 2010, **12**(6):370-375.

13. Qureshi N, Carroll JC, Wilson B, Santaguida P, Allanson J, Brouwers M, Raina P: **The current state of cancer family history collection tools in primary care: a systematic review**. *Genetics in medicine : official journal of the American College of Medical Genetics* 2009, **11**(7):495-506.

14. Wilson BJ, Qureshi N, Santaguida P, Little J, Carroll JC, Allanson J, Raina P: **Systematic review: family history in risk assessment for common diseases**. *Ann Intern Med* 2009, **151**(12):878-885.

15. Census 2006 - 2B (Long Form). Statistics Canada AJ, 2012, at <http://www23.statcan.gc.ca/imdb-bmdi/pub/instrument/3901_Q2_V3-eng.pdf.)>.
