# Supplementary material for: Developing clinical decision tools to implement chronic disease prevention and screening in primary care: the BETTER 2 program (building on existing tools to improve chronic disease prevention and screening in primary care)
Source: Implement Sci. 2015 Aug 4;10:107. doi: 10.1186/s13012-015-0299-9 (PMC4523915; doi:10.1186/s13012-015-0299-9)
Supplement: Additional file 3: — CDPS care maps. (PDF 184 kb) [file 13012_2015_299_MOESM3_ESM.pdf]

The BETTER Chronic Disease Primary Prevention and Screening Map

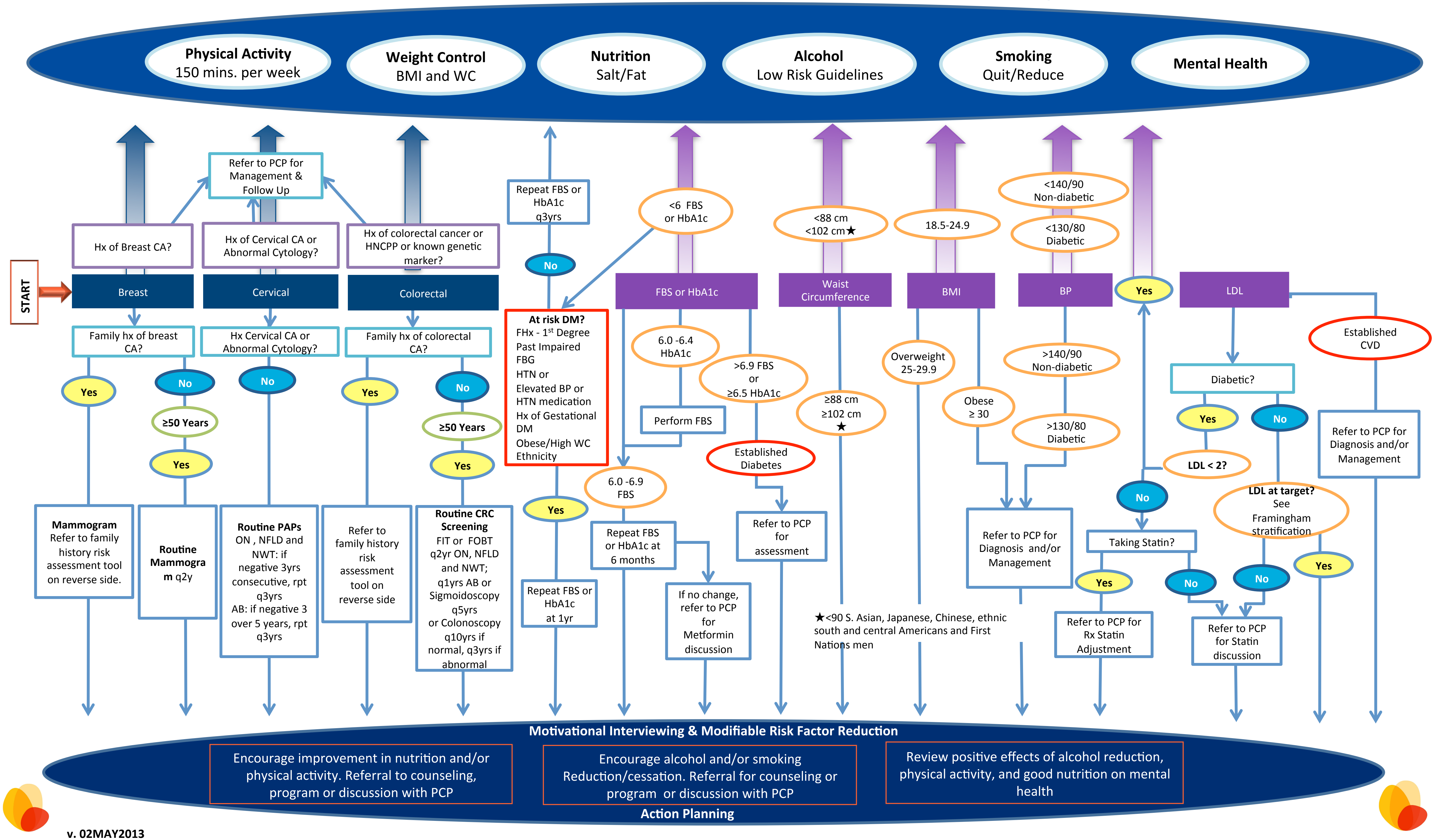

v. 02MAY2013

Framingham Risk Stratification

Risk Level	Initiate treatment if:	Primary treatment target: LDL-C
**HIGH**<br>(10-year CVD risk ≥ 20%)	CAD, PVD, Atherosclerosis*, Most patients with diabetes**	< 2.0 mm/L<br>***or***<br>50% ↓ LDL-C
**MODERATE**<br>(10-year CVD risk 10-19%)	LDL-c > 3.5 mmol/L<br>***or***<br>TC/HDL-C > 5.0<br>***or***<br>hsCRP > 2 mg/L in men >50 years and women >60 years	< 2.0 mm/L<br>***or***<br>50% ↓ LDL-C
**LOW**<br>(10-year CVD risk < 10%)	LDL-C<br>≥ 5.0 mmol/L	50% ↓ LDL-C

Note: In patients with a family history of CVD in a first-degree relative before age 60, the calculated 10-year CVD risk should be **multiplied by 2**.

*Evidence of atherosclerosis = vascular bruits, ABI <0.9, documented CAD, CVA, (TIA or evidence of carotid disease) or peripheral vascular disease
**In men > 45 years, women >50 years with diabetes, as well as some younger people with diabetes who have additional risk as per CDA guidelines.

Source: Pfizer Inc., Cardiovascular Risk Assessment. CA0109LI024E.

Family History (FH) Risk Assessment Tool

Disease	Elevated Risk	Screening Action
**Breast Cancer (BC)**	≥ 2 cases of BC on same side of family, especially:<br>• In closely related relatives<br>• In more than one generation<br>• When BC is diagnosed < age 50<br>***or***<br>Any case of ovarian cancer ***or***<br>Bilateral BC ***or***<br>BC in male relative ***or***<br>BC age < 60 in Ashkenazi Jewish women ***or***<br>Identified BRCA1 or BRCA2 mutation in a 1st degree relative and patient has not had genetic testing	Consider referral to a genetics clinic.<br><br>Annual screening with MRI in addition to mammography starting at age 30
**Colorectal Cancer (CRC)**	Any family history of:<br>CRC ***or***<br>Multiple cancers (CRC or associated cancers) ***or***<br>Cancers at a young age (< 50, particularly if <35)<br>**OR**<br>Personal history of inflammatory Bowel Disease (chronic ulcerative colitis or Crohn's disease)	One 1st degree relative with CRC > 60 ***or*** ≥ two 2nd degree relatives with CRC ***or*** One 2nd/3rd degree relative with CRC – FOBT or FIT q2 years.<br><br>One 1st degree relative with CRC < 60 ***or*** ≥ two 1st degree relatives with CRC any age – Colonoscopy q5 years beginning age 40 or 10 years earlier then youngest dx of cancer, whichever comes first.<br><br>One 1st degree relative with CRC < 50 – consider referral to genetics<br><br>Personal history of inflammatory bowel disease – Colonoscopy beginning 8-10 years after diagnosis
**CHD**	1st degree relative diagnosed with CHD* age < 60	No specific action, modifies Framingham
**Diabetes**	1st degree relative	q1 year

***CHD = angina, MI and CHF.**
