# Supplementary material for: Developing clinical decision tools to implement chronic disease prevention and screening in primary care: the BETTER 2 program (building on existing tools to improve chronic disease prevention and screening in primary care)
Source: Implement Sci. 2015 Aug 4;10:107. doi: 10.1186/s13012-015-0299-9 (PMC4523915; doi:10.1186/s13012-015-0299-9)
Supplement: Additional file 4: — Prevention visit form. (PDF 494 kb) [file 13012_2015_299_MOESM4_ESM.pdf]

PREVENTION VISIT FORM

PATIENT INITIALS: ___ ___ ___

Record the most recent details in each section as recorded on the patient's chart/record or Health Survey that occurred:

- Between the date of the previous Prevention Visit (date 1) and the date of the current Prevention Visit (date 2) OR
- If this is the patient's first Prevention Visit (date 2), record details that occurred up to 2 years prior to the Prevention Visit date (date 1), unless otherwise specified by a question.
- **Complete Sections A1 and B-J prior to the visit and Sections A2, K and L during the visit.**

Today's DATE: ___|___ / ___|___|___ / __|__|__|__ (dd mmm yyyy)

Type of Visit: ❑ In-person ❑ Telephone ❑ Video

DATE RANGE

DATE 1: ___|___ / ___|___|___ / __|__|__|__ (dd mmm yyyy) ***TO*** DATE 2: ___|___ / ___|___|___ / __|__|__|__ (dd mmm yyyy)

SECTION A1: General Patient Information (As per chart)

	Not Recorded	Recorded *Details recorded in chart*	Date Recorded in chart dd/mmm/yyyy	Requires PP Follow-up
1. Gender:	____	_____Male _____Female		
2. Date of Birth:	____	___|___ / ___|___|___ / __|__|__|__ (dd mmm yyyy)		
3. Ethnicity	____	________________________________		
4. BMI:	____	_______._______kg/m^2	___|___ / ___|___|___ / __|__|__|__	❑ No ❑ Yes
5. Waist Circumference:	____	_____.__cm OR _____.__inches	___|___ / ___|___|___ / __|__|__|__	❑ No ❑ Yes
6. Blood Pressure: (last 12 months)	____	a. ________/_______ systolic / diastolic	___|___ / ___|___|___ / __|__|__|__	❑ No ❑ Yes

SECTION A2: General Patient Information (As per Prevention Visit)

	Measurement	Date Measured dd/mmm/yyyy	Requires PP Follow-up
1. Height:	_____.___cm OR _____ft ____.___ inches	___|___ / ___|___|___ / __|__|__|__	
2. Weight:	_______.___lbs OR _______.___kg	___|___ / ___|___|___ / __|__|__|__	
3. BMI:	_______._______kg/m^2	___|___ / ___|___|___ / __|__|__|__	❑ No ❑ Yes
4. Waist Circumference:	_____.__cm OR _____.__inches	___|___ / ___|___|___ / __|__|__|__	❑ No ❑ Yes
5. Blood Pressure:	________/_______ systolic / diastolic	___|___ / ___|___|___ / __|__|__|__	❑ No ❑ Yes

PATIENT INITIALS: ___ ___ ___

SECTION B: Lab Results *(Record most recent value within date range)*

	Not Recorded	Recorded *Details recorded in chart*	Date of Lab Results dd/mmm/yyyy	Requires PP Follow-up
1. Fasting Blood Sugar: *(in past 3 years)*	______	a. ________.______ mmol/L	___|___ / ___|___|___ / __|__|__|__	❑ No ❑ Yes
2. HbA1c:	______	a. ________.______%	___|___ / ___|___|___ / __|__|__|__	❑ No ❑ Yes
3. Total Cholesterol:	______	a. ________.______ mmol/L	___|___ / ___|___|___ / __|__|__|__	❑ No ❑ Yes
4. HDL:	______	a. ________.______ mmol/L	___|___ / ___|___|___ / __|__|__|__	❑ No ❑ Yes
5. LDL:	______	a. ________.______ mmol/L	___|___ / ___|___|___ / __|__|__|__	❑ No ❑ Yes

SECTION C: Screening Tests *(**If first visit**, past 2 years except for: Pap Smear – past 3, Sigmoidoscopy – past 5 and Colonoscopy – past 10 years)*

***If patient is <50 yo**, colorectal cancer screen is not applicable. **If patient is <50 yo and female**, breast cancer screen is not applicable.*

Screening Test	a. Test Completed				If RECORDED and COMPLETED: Date most recent Completed			Requires PP Follow-up
	Test Not Applicable	*Not Recorded*	Recorded: Not completed	Recorded: Completed	DD	MMM	YYYY	
1. FOBT/FIT								❑ No ❑ Yes
2. Sigmoidoscopy								❑ No ❑ Yes
3. Colonoscopy								❑ No ❑ Yes
4. Pap Smear								❑ No ❑ Yes
5. Mammogram								❑ No ❑ Yes

SECTION D: Health Conditions *(**If patient male** do not record details for breast, cervical & ovarian cancer)*

Do not complete this section for follow-up visits.

Health Condition	Patient has health condition				If RECORDED: Age Diagnosed	Eligible for Screen
	Not Applicable	Not Recorded	Recorded: No Condition	Recorded: Condition		
1a. Diabetes						❑ No ❑ Yes
1b. Gestational						
1c. Impaired FBS/glucose						
2. Cardiovascular Disease (angina, congestive heart failure, heart attack, coronary artery disease)						❑ No ❑ Yes
3. Breast Cancer *if Condition complete 3a*						❑ No ❑ Yes
3a. Breast Cancer type 1 (BRCA1) or Breast Cancer type 2 (BRCA2) gene mutation						
4. Cervical Cancer						❑ No ❑ Yes
5. Colorectal Cancer *If Condition complete 5a*						❑ No ❑ Yes

PATIENT INITIALS: ___ ___ ___

Health Condition	Patient has health condition: Not Applicable	Not Recorded	Recorded: No Condition	Recorded: Condition	If RECORDED: Age Diagnosed	Eligible for Screen
5a. Polyps						
6. High Cholesterol (Hyperlipidemia, lipidemia)						❑ No ❑ Yes
7. Hypertension						❑ No ❑ Yes
8. Ovarian Cancer						❑ No ❑ Yes

SECTION E: Patient Self-Reported Family History Risk *(Source of information is Health Survey)*

Requires PP Follow-up

Is the patient at increased familial risk for:

		Requires PP Follow-up
1. Diabetes?	❑ No ❑ Yes	❑ No ❑ Yes
2. Breast Cancer?	❑ No ❑ Yes	❑ No ❑ Yes
3. Colorectal/Bowel Cancer?	❑ No ❑ Yes	❑ No ❑ Yes
4. Cardiovascular/Heart Disease?	❑ No ❑ Yes	❑ No ❑ Yes

SECTION F: Framingham Risk Assessment *(Record value calculated if able)*

	Not Available	Value From Chart	Value From Visit	Risk Value	Date Risk Assessment Completed dd/mmm/yyyy	Requires PP Follow-up
1. Framingham Risk:	_____	_____	_____	_________._____ %	____|___ / ___|___|___ / __|__|__|__	❑ No ❑ Yes

SECTION G: Mental Health Screen *(Source of information is Health Survey)*

Requires PP Follow-up

1. Patient Health Questionnaire 2 question screen for depression positive? ❑ No ❑ Yes — Requires PP Follow-up: ❑ No ❑ Yes

SECTION H: Lifestyle *(Sources of information are the patient's chart and Health Survey. If date details recorded unknown, still record details)*

Requires PP Follow-up

1. Is information regarding the patient's alcohol consumption recorded in their chart? ❑ No ❑ Yes
   a. Is the patient identified as a binge drinker? (Health Survey) ❑ No ❑ Yes — Requires PP Follow-up: ❑ No ❑ Yes
   b. What is the patient's alcohol consumption? (Health Survey)
      b1. Number of drinks _______ PER ❑ Day ❑ Week ❑ Month — Requires PP Follow-up: ❑ No ❑ Yes
2. AUDIT-C alcohol screen positive? (Health Survey) ❑ No ❑ Yes — Requires PP Follow-up: ❑ No ❑ Yes
3. Is patient smoking status recorded in their chart? ❑ No ❑ Yes→ specify below
   a. What is the patient's smoking status? (Chart) ❑ Non-smoker ❑ Ex-smoker ❑ Current Smoker ❑ Quitting
   b. How many cigarettes does the patient smoke? (Health Survey) _______ PER ❑ Day ❑ Week — Requires PP Follow-up: ❑ No ❑ Yes

PREVENTION VISIT FORM

PATIENT INITIALS: ___ ___ ___

Requires PP Follow-up

4. Are details regarding the patient's physical activity recorded in their chart? ❑ No ❑ Yes

   a. Number of minutes of exercise per week? (Health Survey) __________ minutes per week ❑ No ❑ Yes

5. General Practice Physical Activity Questionnaire score (Health Survey) ________________________ ❑ No ❑ Yes

6. Are details regarding the patients diet/nutrition recorded their chart? ❑ No ❑ Yes

7. Starting the Conversation Nutrition Questionnaire score (Health Survey) ____________ ❑ No ❑ Yes

SECTION I: Current Medications *(Indicate whether the patient was prescribed medications for the following within date range)*

**Medications**	**a. Currently Prescribed**	
	**No**	**Yes**
1.Blood Pressure		
2. Diabetes		
3. Cholesterol		
4. Smoking Cessation		
5. Alcohol Cessation		
6. Mental Health (ex. depression, anxiety, psychosomatic disorders)		

SECTION J: Name of Primary Care Provider and/or Clinic *(Record information for the healthcare provider(s) responsible for the patient's ongoing medical care and the care that they provide)*

Primary Care Provider: ____________________

Clinic: ____________________

Type of Medical Care: ____________________

____

Primary Care Provider: ____________________

Clinic: ____________________

Type of Medical Care: ____________________

____

PATIENT INITIALS: ___ ___ ___

SECTION K: Referrals or Discussions arranged with Health Professionals (FP, PP, Nurse Practitioner, Dietician, Nutritionist, Pharmacist), Specialists or Programs *(To be **completed after** Prevention Visit with patient)*

Referral or Discussion Regarding:	a. Referral			b. Referral to			c. Referral Details			Requires PP Follow-up
	Offered	Refused	Accepted or Patient Initiated	Primary Care Provider (e.g. FP)	Internal resource/ program	External resource/ program	In Progress	Completed	Not Completed	
1.Cardiology/Heart Disease										❑ No ❑ Yes
2. Diabetes										❑ No ❑ Yes
3. Hypertension										❑ No ❑ Yes
4. Cholesterol										❑ No ❑ Yes
5. Nutrition/Diet										❑ No ❑ Yes
6. Weight control										❑ No ❑ Yes
7. Physical activity or exercise										❑ No ❑ Yes
8. Alcohol cessation										❑ No ❑ Yes
9. Smoking cessation										❑ No ❑ Yes

SECTION L: Notes *(Consider capturing information of programs patients have referred themselves to. In particular, those that may become resources to the practice)*

____

____

____

____

____

____

____

Time spent administering Health Survey to patient (mins.): ____________

Time spent completing this form to prepare for Prevention Visit (mins.): ____________

Time spent on Prevention Visit (mins.): ____________

Signature______________________________ **Date Signed (dd-mmm-yyyy):** ___|___ / ___|___|___ / __|__|__|__
