# Supplementary material for: Developing clinical decision tools to implement chronic disease prevention and screening in primary care: the BETTER 2 program (building on existing tools to improve chronic disease prevention and screening in primary care)
Source: Implement Sci. 2015 Aug 4;10:107. doi: 10.1186/s13012-015-0299-9 (PMC4523915; doi:10.1186/s13012-015-0299-9)
Supplement: Additional file 5: — The Bubble diagram. (PDF 934 kb) [file 13012_2015_299_MOESM5_ESM.pdf]

Chronic Diseases We Will Focus on During Your Prevention Visit

Cancer

- **Colorectal Cancer**
  Fecal occult blood test (FOBT) or Fecal Immunochemical test (FIT)
  Sigmoidoscopy
  Colonoscopy

Diabetes

- Fasting Blood Sugar every 3 years, < 6 mmol

OR

- HbA1c every 3 years, <6.0%
- High risk – HbA1c or FBS every 1 year

Heart Disease

- BP ≤140/90 (Non-Diabetic), Framingham risk score <10%
- BP ≤130/80 (Diabetic), UKPDS score
- LDL (Diabetic): < 2 mmol/L
- LDL (Non-Diabetic):
  - <3.5 mmol/L (moderate risk)
  - <5 mmol/L (low risk)

These are regular screening intervals and healthy targets

Family History

Male

Mental Health

Nutrition

- Less than 1 tsp of salt each day
- Limit high fat foods

Physical Activity

- Engage in 150 minutes (cumulative) of moderate physical activity each week

- Normal body mass index 18.5-24.9
- Waist circumference <102cm

Alcohol

- Low-risk drinking guidelines: 1-2 drinks a day, total 14 drinks each week

1 drink = 1 beer, 5 oz wine or 1.5 oz liquor

Smoking

- Set a quit date
- Plan to reduce

Factors that Determine Your Risk for Chronic Disease

v.02MAY2013

THE BETTER PROGRAM

Chronic Diseases We Will Focus on During Your Prevention Visit

Cancer

- **Pap Test** every 1-3 years to screen for cervical cancer
- **Mammogram** every 2 years to screen for breast cancer
- **Colorectal cancer**

  Fecal occult blood test (FOBT) or Fecal Immunochemical test (FIT)
  Sigmoidoscopy
  Colonoscopy

Diabetes

- Fasting Blood Sugar every 3 years, < 6 mmol

OR

- HbA1c every 3 years, <6.0%
- High risk – HbA1c or FBS every 1 year

Heart Disease

- BP ≤140/90 (Non-Diabetic), Framingham risk score <10%
- BP ≤130/80 (Diabetic), UKPDS score
- LDL (Diabetic): < 2 mmol/L
- LDL (Non-Diabetic):
  - <3.5 mmol/L (moderate risk)
  - <5 mmol/L (low risk)

These are regular screening intervals and healthy targets

Family History

Female

Mental Health

Nutrition

- Less than 1 tsp of salt each day
- Limit high fat

- Normal body mass index 18.5-24.9
- Waist circumference <88cm

Physical Activity

- Engage in 150 minutes (cumulative) of moderate physical activity each week

Alcohol

- Low-risk drinking guidelines: 1 drinks a day, total 7 drinks each week

1 drink = 1 beer, 5 oz wine or 1.5 oz liquor

Smoking

- Set a quit date
- Plan to reduce

Factors that Determine Your Risk for Chronic Disease

v.02MAY2013

THE BETTER PROGRAM
