# Supplementary material for: Developing clinical decision tools to implement chronic disease prevention and screening in primary care: the BETTER 2 program (building on existing tools to improve chronic disease prevention and screening in primary care)
Source: Implement Sci. 2015 Aug 4;10:107. doi: 10.1186/s13012-015-0299-9 (PMC4523915; doi:10.1186/s13012-015-0299-9)
Supplement: Additional file 6: — The prevention prescription. (PDF 354 kb) [file 13012_2015_299_MOESM6_ESM.pdf]

THE BETTER PROGRAM

Date: _____/_____/_____ (month) (day) (year) **Your Initials:** _____/_____/_____

Your Health Care Team and You Working Together:
THE PREVENTION PRESCRIPTION

At your visit, we worked together to identify a number of important actions you can take to help prevent chronic disease. **This tool can be used to increase your understanding of the recommended guidelines for regular screening around some of the following potential lifestyle concerns and chronic diseases. Together, we can take steps to support and improve your health and well-being!**

Screening For:	Your Status/Results	When to Re-Check	Referral's/Actions
Cardiovascular Disease			
BMI			
WC			
Blood pressure			
Cholesterol			
Diabetes			
FBS/HbA1c			
Cancer Screening			
FOBT/FIT			
Sigmoidoscopy			
Colonoscopy			
Pap test			
Mammogram			
Lifestyle Concerns			
Physical activity			
Diet			
Alcohol			
Smoking			
Other lifestyle concerns:			

Resources available to help you (websites, handouts etc.):

Your next prevention appointment is in ___ months with: ____________________

Your health care provider's signature: ____________________

THE BETTER PROGRAM

Date:_____/_____/_____ Your Initials:_____/_____/_____
(month) (day) (year)

	1	2	3	4	5	6	7	
	**WAYS I CAN IMPROVE MY HEALTH – WHAT? (Set Your Goal)**	**WHAT WILL STOP YOU?**	**HOW MUCH?**	**HOW OFTEN?**	**WHEN?**	**WHERE?**	**RATE YOUR CONFIDENCE (Choose One per Goal)**	
**Goal #1**							*How Confident Am I That I Can Reach This Goal?*	❍ 0 – Not at all confident<br>❍ 1<br>❍ 2<br>❍ 3 – A little confident<br>❍ 4<br>❍ 5 – Somewhat confident<br>❍ 6<br>❍ 7<br>❍ 8 – Very confident<br>❍ 9<br>❍ 10 – Totally confident
**Goal #2**							*How Confident Am I That I Can Reach This Goal?*	❍ 0 – Not at all confident<br>❍ 1<br>❍ 2<br>❍ 3 – A little confident<br>❍ 4<br>❍ 5 – Somewhat confident<br>❍ 6<br>❍ 7<br>❍ 8 – Very confident<br>❍ 9<br>❍ 10 – Totally confident
**Goal #3**							*How Confident Am I That I Can Reach This Goal?*	❍ 0 – Not at all confident<br>❍ 1<br>❍ 2<br>❍ 3 – A little confident<br>❍ 4<br>❍ 5 – Somewhat confident<br>❍ 6<br>❍ 7<br>❍ 8 – Very confident<br>❍ 9<br>❍ 10 – Totally confident

v. 03MARCH2013
